# Intra-Vitreal Administration of Microvesicles Derived from Human Adipose-Derived Multipotent Stromal Cells Improves Retinal Functionality in Dogs with Retinal Degeneration

**DOI:** 10.3390/jcm8040510

**Published:** 2019-04-13

**Authors:** Anna Cislo-Pakuluk, Agnieszka Smieszek, Natalia Kucharczyk, Peter G.C. Bedford, Krzysztof Marycz

**Affiliations:** 1 Trzebnicka Veterinary Clinic, Department of Ophthalmology, Kosciuszki 18 St, 55-100 Trzebnica, Poland; ocuvet@gmail.com (A.C.-P.); natalia.nestorowicz@gmail.com (N.K.); 2 Viva Veterinary Clinic, Department of Ophthalmology, Strachocinska 143 St., 51-518 Wroclaw, Poland; 3 Department of Experimental Biology, The Faculty of Biology and Animal Science, University of Environmental and Life Sciences, 50-375 Wroclaw, Poland; agnieszka.smieszek@upwr.edu.pl; 4 International Institute of Translational Medicine, Jesionowa 11 St, 55-124 Malin, Poland; 5 Royal Veterinary College, London NW1 0TU, UK; profg1@btinternet.com; 6 Ophthalmology Referrals, 25, Great North Road, Brookmans Park, Herts., North Mymms, Hatfield AL9 6LB, UK; 7 Faculty of Veterinary Medicine, Equine Clinic-Equine Surgery, Justus-Liebig-University, 35392 Gießen, Germany

**Keywords:** microvesicles, human multipotent stromal cells, retinal regeneration

## Abstract

This study was designed to determine the influence of microvesicles (MVs) derived from multipotent stromal cells isolated from human adipose tissue (hASCs) on retinal functionality in dogs with various types of retinal degeneration. The biological properties of hASC-MVs were first determined using an in vitro model of retinal Muller-like cells (CaMLCs). The in vitro assays included analysis of hASC-MVs influence on cell viability and metabolism. Brain-derived neurotrophic factor (BDNF) expression was also determined. Evaluation of the hASC-MVs was performed under normal and oxidative stress conditions. Preliminary clinical studies were performed on ten dogs with retinal degeneration. The clinical studies included behavioral tests, fundoscopy and electroretinography before and after hASC-MVs intra-vitreal injection. The in vitro study showed that CaMLCs treated with hASC-MVs were characterized by improved viability and mitochondrial potential, both under normal and oxidative stress conditions. Additionally, hASC-MVs under oxidative stress conditions reduced the number of senescence-associated markers, correlating with the increased expression of BDNF. The preliminary clinical study showed that the intra-vitreal administration of hASC-MVs significantly improved the dogs’ general behavior and tracking ability. Furthermore, fundoscopy demonstrated that the retinal blood vessels appeared to be less attenuated, and electroretinography using HMsERG demonstrated an increase in a- and b-wave amplitude after treatment. These results shed promising light on the application of cell-free therapies in veterinary medicine for retinal degenerative disorders treatment.

## 1. Introduction

The etiology of retinal degeneration in dogs is complex, encompassing both acquired and genetically determined diseases. Amongst the many causes, acquired retinal degeneration could be due to infectious disease, trauma, toxicity elevated intraocular pressure and autoimmune factors. Degeneration can be slow or sudden in onset. For instance, in sudden acquired retinal degeneration (SARD) the onset of photoreceptor degeneration is usually very rapid, whereas the progress of an acquired retinopathy could vary depending on cause. Some lesions are stable and focal whereas in other situations progression to generalized degeneration can be noted [1]. The genetically determined retinopathies vary in etiology, rate of progression and age of onset. Progressive retinal atrophy (PRA) is the collective term used for several clinically and genetically heterogenous canine hereditary retinal dystrophies [1,2,3]. Most PRA is usually characterized initially by reduced vision in low levels of illumination due to rod dysfunction, but later total blindness occurs as the result of subsequent cone degeneration [3,4]. Despite the diversity in the etiology of PRA, there are similarities in the ophthalmoscopic appearance of the retina towards the end of the degenerative process. The main features are tapetal hyperreflectivity, vascular attenuation, atrophy of the optic nerve head and pigmentary changes [1]. Deterioration of the retina causes a loss of function which can be detected by electroretinographic investigation (ERG) even before the ophthalmoscopic features become visible [5,6]. PRA is considered to be of significant health concern, occurring in over one hundred dog breeds [1,3]. Oxidative stress may play an important role in the pathogenesis of PRA and it has been reported that the cone degeneration in human retinitis pigmentosa (RP) is related to accumulation of reactive oxygen species (ROS) and markedly elevated oxygen levels have been observed in the retina after rod degeneration [7,8]. Moreover, antioxidants have been shown to decrease cone death in several animal models of retinal degeneration [8,9,10] and recently it has been proposed that nutritional antioxidant supplementation could improve the visual function of dogs with retinal degeneration [11]. Several substances that inhibit photoreceptor loss in animals have been reported, including nerve growth factor (NGF) and brain-derived neurotrophic factor (BDNF) [12,13]. BDNF has also been recognized as a promotor of neural stem progenitor cells proliferation through activation of AKT (protein kinase B) signaling [12]. Beside this neurostimulatory role, Mey and Thanos demonstrated that brain-derived neurotrophic factor (BDNF) plays a neuroprotective role in an experimental rat model for retinal degeneration [14]. In this model intra-vitreal administered BDNF has been shown to improve the process of retinal regeneration. Moreover promising data was presented by Domenici et al. (2014) who showed that the intravitreal injection and topical instillation of exogenous BDNF in Dilute Brown Non-Agouti mice strain (DBA/2J) offer protection for retinal ganglion cells [15]. Nevertheless the therapeutic use of BDNF may be a distant prospect, due to its low penetrability.

Recently, considerable attention has been paid to the role of mesenchymal stromal cells (MSCs) as a possible treatment for retinal disorders. Progenitor cells are found not only in bone marrow and adipose tissue, but also in cartilage, muscle, lipid, myocardial cells, glial cells and neurons [16]. Currently, adipose-derived stromal cells (ASCs) are considered to be the most suitable source of MSCs, mainly due to their highly proliferative activity and immunomodulatory properties, but also due to the safe method of their isolation [17]. Recently, it has been reported that ASCs exhibit a neuroprotective effect by secreting trophic/neuroregulatory factors [17,18]. The pro-regenerative potential of ASCs is explained by their ability to produce and secrete nano- and micro-sized extracellular vehicles (EVs), which include both exosome and microvesicles (MVs) populations [19,20]. Microvesicles are classified as heterogeneous population of particles released from membrane plasma, measuring between 30nm to 1μm in diameter. They have also been shown to play an important role in intercellular signaling and they possess an ability to transfer coding and non-coding RNAs (ncRNAs), including both microRNAs and long non-coding RNAs (miRNAs, lncRNAs) [21,22,23]. There is a growing interest in the development of cell-free therapies based on the use of MVs derived from progenitor cells. Mounting evidence indicates that the use of MVs represents a novel possible therapeutic tool for tissue regeneration, especially in degenerative disorders. The MVs stimulate angiogenesis, stem cell differentiation, cell migration and the activation of anti-apoptotic mechanisms. These cellular processes are crucial in terms of the amelioration of the clinical signs seen in many retinal degenerations, including PRA and the acquired retinopathies [19,20,21,23]. Recently, it has been demonstrated that embryonic stem cell derived MVs are able to induce de-differentiation and pluripotency in Müller cells, activities which may result in the early retinogenic differentiation of those cells [24]. Müller cells are analogous to radial glia in the retina and are recognized as progenitor retinal cells. They possess the ability to differentiate within multiple retinal lineages, producing photoreceptors and inner retinal neurons, thus making them potentially useful for retinal regeneration research [25,26,27,28]. Bearing in mind the positive effect of MVs on Müller cell differentiation, we were interested in determining whether MVs influence the proliferative activity, apoptosis, senescence and mitochondrial potential of those cells and to test if this pro-regenerative effect of MVs may be related to BDNF production and the activation of mTOR (mammalian target of rapamycin kinase), Akt and PI3K (phosphoinositide 3-kinases) pathways. The biological properties of human adipose-derived multipotent stromal cell-MVs (hASC-MVs) were evaluated using canine Müller-like cells (CaMLCs) and the effect of clinically administrated hASC-MVs in dogs with retinal degeneration of different etiologies was assessed.

## 2. Materials and Methods 

### 2.1. The Preparation of Conditioned Medium from Human Adipose-Derived Multipotent Stromal Cells (CM-ASC) Rich in Microvesicles (MVs)

The cultures of hASCs used for the procedure have been previously characterized and described [29]. For this study, cells with an immunophenotype confirming their mesenchymal origin were used. Their multipotent properties were recognized, and their potential for differentiation into adipocytes, chondrocytes and osteocytes has been established [29,30]. The cells were cultured in Dulbecco’s Modified Eagle’s Medium (DMEM)/Ham’s F12 medium, supplemented with 10% fetal bovine serum (FBS) and with a 1% antibiotic-antimycotic solution, referred to as a complete growth medium for hASCs (CGM-hASCs). The cultures of hASCs were maintained in a CO_2_ incubator (37 °C and 5% CO_2_) and passaged three times with the trypsin solution TrypLE™ Express (Thermo Fisher Scientific, Warszawa, Poland). The CGM-hASCs was changed every two days. For the preparation of the conditioned medium (CM-hASCs), the cells at passage three (p3) were labeled with a PKH67 Fluorescent Cell Linker Kit (Sigma Aldrich, Poznan, Poland) and cultured to obtain 100% confluency. At high confluency the CGM-hASCs were removed and the cultures were washed three times using Hanks’ Balanced Salt Solution (HBSS) without the calcium and magnesium. The cultures were then serum starved for 48 h in a starvation medium (SM) composed of CGM-hASCs without the serum supplementation. After 48 h, SM was collected and centrifuged initially for 10 min at 300× *g*. The supernatants were then collected, centrifuged again for 100 min at 2000× *g* to remove cell debris and centrifuged in an Amicon Ultra 15 filter (3000 MWCO, Merck, Poznan, Poland) at 5000× *g* for 40 min at 4 °C to obtain the conditioned medium rich in microvesicles (hASC-MVs). The supernatant was centrifuged at 20,000× *g* for 30 min at 4 °C in order to obtain the MVs, then removed and the remaining pellet re-suspended in HBSS. The MVs from the hASCs were stored at −80 °C. Total protein concentration in hASC-MVs was measured using the Bicinchoninic Acid (BCA) Kit Assay Kit (Sigma Aldrich, Poznan, Poland).

### 2.2. Isolation and Characterization of Canine Müller-Like Cells (CaMLC)

Retina derived from a 24 h old female German Shepherd Dog fetus was used for the isolation of primary canine Müller-like cells (CaMLC). Isolated retinal tissue was washed twice using phosphate-buffered saline (PBS), macerated with a sterile scalpel and homogenized using a sterile pestle. The homogenates were filtrated using Falcon^®^ 70 µm cell strainers (Corning, Biokom, Janki, Poland), washed with HBSS and then centrifuged at 300× *g* for 4 min at room temperature. The pellets were then suspended in a culture medium consisting of Dulbecco’s Modified Eagle’s Medium (DMEM)/Ham’s F12 medium, supplemented with 10% FBS and a 2% antibiotic-antimycotic solution (CGM-pCaMLC). The CaMLC were cultured at 37 °C in humidified 5% CO_2_, the medium being replaced every two days. Primary cultures were passaged using a trypsin solution (TrypLE™ Express, Thermo Fisher Scientific, Warszawa, Poland) after two days. Secondary cultures were maintained in Dulbecco’s Modified Eagle’s Medium (DMEM), supplemented with 10% FBS and 1% antibiotic-antimycotic solution (CGM-pCaMLC). The secondary cultures were passaged at around 80% confluency, usually every 3 days. Before this occurred, the experiment cultures of CaMLC were passaged three times (p3). The morphology of cultures was monitored using inverted phase-contrast microscope (Axio Observer A.1, Zeiss, Oberkochen, Germany). The characteristic phenotype of Müller-like cells was determined using reverse transcription–quantitative polymerase chain reaction (RT-qPCR), the details of which are described in Section 2.4.

### 2.3. The Evaluation of the Biological Activity of the Microvesicles Derived from hASCs in Cultures with CaMLCs under Oxidative Stress Conditions

For the experiment CaMLCs (p3) were inoculated into a 24-well plate at a concentration equal to 40,000 cells per well. In order to determine their biological activity, the cells were treated with 200 μM of H_2_O_2_ in CGM-sCaMLC for 1 h in a 5% CO_2_ incubator at 37 °C. After incubation the cells were washed with HBSS and then incubated for a further 24 h with hASC-MVs in a concentration corresponding to the 25 µg/mL of total protein. A comprehensive assessment of cell health state was then completed, the analysis including evaluation of the apoptosis profile in tested cultures and the mitochondrial membrane potential of the CaMLC. Measurements were performed using the Muse™ Cell Analyzer after cell staining with the Muse^®^ Annexin V and Dead Cell Assay Kit and the Muse^®^ Mitopotential Assay Kit (Merck, Warszawa, Poland). The measurements were repeated at least three times. Further, mitochondria were visualized using MitoRed staining completed according to established protocol [31,32]. Also, cultures were counterstained using diamidino-2-phenylindole (DAPI; 1:1000). The analysis of metabolic activity was performed using an Alamar blue assay as described previously [33]. The activity of senescence-associated β-galactosidase (β-gal) in cultures with hASC-MVs, both in normal and oxidative stress conditions, was also determined. For this purpose, cultures were stained with Senescence Cells Histochemical Staining Kit (Sigma Aldrich, Poznan, Poland), the blue stained positive cells examined using an inverted microscope (Axio Observer A.1, Zeiss, Oberkochen, Germany) and analyzed with an ImageJ Pixel Counter [31].

The western blot technique was used for the detection of BDNF in cell homogenates obtained after the experiment. For the measurement, CaMLCs cultures were washed with Hanks’ Balanced Salt solution (HBSS) and lysed with ice-cold RIPA extraction buffer supplemented with protease and phosphatase inhibitors. The protein concentration was determined using the BCA Assay Kit (Merck, Poznan, Poland). The western blot was completed using the Mini Trans-Blot^®^ system (Bio-Rad Poland Sp. z.o.o., Warszawa, Poland). Electrophoresis was performed from samples with equal amounts of protein (30 μg). SDS-PAGE and the protein transfer were completed using protocols described previously [34,35]. Primary antibodies against BDNF (SAB2108004, Sigma Aldrich, Poznan, Poland) were prepared at a dilution of 1:250 in Tris/NaCl/Tween buffer (TBST). The secondary antibody used in the reaction was conjugated with alkaline phosphatase (A9919, Sigma Aldrich, Poznan, Poland). This antibody was prepared at 1:1000 in TBST. The membranes were developed using BCIP^®^/NBT-Purple Liquid Substrate for 10 min. The reaction was stopped by washing the membrane with distilled water. The signals were captured using the Bio-Rad ChemiDoc™ XRS system and analyzed using Image Lab™ Software (version 5.2., Bio-Rad Poland Sp. z.o.o., Warszawa, Poland).

### 2.4. The Reverse Transcription–Quantitative Polymerase Chain Reaction (RT-qPCR)

The mRNA level of the transcripts was evaluated using RT-qPCR. The cells were homogenized using 0.5 mL of TRI Reagent^®^ (Sigma Aldrich, Poznan, Polska) and *total*RNA was isolated using the Chomczynski and Sacchi method [36]. After *total*RNA isolation, genomic DNA residues were removed using the Precision^TM^DNase kit (Primerdesign Ltd., Cambridge, United Kingdom). The cDNA synthesis was completed using 0.5 µg of RNA with the Tetro cDNA Synthesis Kit (Bioline Reagents Ltd., London, United Kingdom). Oligo (dT) primers18 were used during the reaction. The quantitative PCR was performed in a total volume of 10 μL using SensiFast SYBR & Fluorescein Kit (Bioline Reagents Ltd., London, United Kingdom). The cDNA concentration in the reaction mixture was 1 μL and the specific oligonucleotide concentration was equal to 400 nM. The sequences of primers used in the qPCR are listed in Table S1. The qPCR was performed using the CFX Connect Real-Time PCR Detection System (Bio-Rad Polska Sp. z.o.o., Warszawa, Poland). The gene expression fold change was calculated based on the quantification cycle (Cq) related to the expression of the housekeeping gene, glyceraldehyde-3-phosphate dehydrogenase (GAPDH). Additionally, CaMLCs marker reaction products were analyzed by electrophoresis in 2% agarose gel (Novazym, Poznan, Poland) stained with SYBR™ Safe DNA Gel Stain (Thermo Fisher Scientific, Warszawa, Poland).

### 2.5. The Clinical Cases

#### 2.5.1. Animals

Ten dogs with behavioral changes that were possibly due to sight deterioration were referred for ophthalmic examination between the years 2016–2018. The dogs were aged between 2 to 13 years (8.5 ± 4 years): four animals were pure-bred, six were of mixed breed, six were female and four were male. In seven of the ten dogs, the clinical histories, results of fundoscopy and absence of other possible causes of retinal degeneration rendered a tentative diagnosis of progressive retinal atrophy (PRA) possible. Details of the patients are presented in Table 1. The owner of treated dogs presented written agreement for publishing these data.

#### 2.5.2. Behavioral Observation

Deterioration in sight was the first sign noticed by all the owners, but initially many suspected that the hesitation they saw was due to behavioral reasons. Two dogs were working dogs and their problems were noted as anxiety and nervousness in new places. The dogs started to sniff more intensively, especially after entering a new room or after getting out of a car. The handlers also noticed that the dogs were distracted, had difficulty in assessing distance and were hesitant, especially in dark places and in new surroundings. Other owners had not noticed these behavioral changes, but all commented that vision was affected initially in low levels of illumination and at night. Dazzle and menace reflexes were normal, but problems were noted during maze testing. All dogs were put through the maze in a variety of lighting conditions and noticeable hesitation was observed, especially in dark and dimmed light. Two dogs had post inflammatory retinal degeneration; in the case of the Collie, this was due to a chorioretinitis of unknown etiology, but in the crossbred dog, the chorioretinitis was considered to be associated with a *Borelia sp.* infection, diagnosed twelve months previously. The infection had been confirmed using PCR and, according to the owner, the dog’s vision had remained deficient since then. In both dogs, the owners had noticed the sight problem regardless of the lighting conditions.

#### 2.5.3. Ophthalmic Examination

The ophthalmic examination included the Schirmer tear test, fluorescein staining, slit lamp biomicroscopy, tonometry (Tonopen XL, Reichert Technologies, Depew, NY, USA), ultrasound and fundus examination using both direct and indirect ophthalmoscopy. Fundus changes due to retinal degeneration were present in all dogs. These changes included bilateral blood vessel attenuation, tapetal hyperreflectivity and early optic disc atrophy. In the seven dogs in which PRA was suspected there were no other ocular abnormalities and no history of systemic disease which could cause retinal degeneration. However in the absence of commercially available DNA based tests, genetic testing for PRA was not possible for the breeds involved in this study.

#### 2.5.4. Electroretinography

To assess the rod and cone function before MVs administration (Exam I) electroretinography (ERG) was completed in 9 dogs based on a Short Protocol using a Handheld Multispecies ERG HMsERG- portable mini-Ganzfeld ERG unit (RetVet Corp, Columbia, MO, USA). The procedure was performed accordingly to the guidelines for clinical electroretinography in the dog [37]. The dogs were premedicated intramuscularly with a mixture of dexmedetomidine (Dexdomitor, Orion Pharma, Warsaw, Poland) 0.005 mg/kg, butorphanol 0.15 mg/kg (Butomidor, Orion Pharma, Warsaw, Poland) and midazolam 0.1 mg/kg (Midanium 5 mg/mL, Polfa Warszawa SA, Warsaw, Poland). General anesthesia was induced with intravenous propofol 2 mg/kg propofol 1%, 10 mg/mL (Fresenius Kabi, Bad Homburg vor der Höhe, Germany) and where prolonged general anesthesia was required, intravenous propofol was administered as required at 1 mg/kg. Prior to ERG recording, pupils were maximally dilated with tropicamide (Tropikamidum WZF 1% Polfa Warszawa SA, Warsaw, Poland). Before ERGs were recorded, the baseline and impedance values were verified. The protocol starts at time 0 with Standard Rod & Cone flash intensity (0.00 Hz, 3 cd/m^2^/s). After 60 s of dark adaptation another Standard Rod & Cone flash exam was performed (average of 4 traces, 0.10 Hz, 3 cd/m^2^/s). This was then followed after a 5 min adaptation to darkness by a recording of mixed rod-cone response to a standard flash (average of 4 traces, 0.10 Hz, 3 cd/m^2^/s). The High Intensity Rod-Cone test was assessed after a 2-minute dark adaptation (4 traces, 0.05 Hz, 10 cd/m^2^/s) and the cone flicker test (128 flashes, 30.30 Hz, 3 cd/m^2^/s). To maintain a consistent level of adaptation in bilateral recordings, room lights were turned on for 10 min after the recording of the first eye, and then turned off to begin the protocol of the second eye. A- and b-wave amplitudes were measured from baseline to the first trough and from that trough to next positive peak. Implicit times were measured from stimulus to the positive peaks a- and b-waves. For flicker tests, the frequency domain amplitude of the 1st harmonic was extracted. In the Kelpie after 20 min of dark adaptation Dog Diagnostic Protocol of HMsERG was performed.

#### 2.5.5. The Administration of hASC-MVs

0.1 ml of MVs suspended in HBSS and containing 25 µg/mL of total proteins was injected intravitreally into one eye of each dog included in the study. The bulbar conjunctiva of the treated eye was flushed with 3% boric acid (Borasol, 3%, 100 g, Prolab S, Nakło nad Notecią, Poland) and 0.5% solution of jodpovidone (Braunol 10%, Braun, Melsungen, Germany). Corneoscleral forceps were used to stabilize the eye and a 23 G needle was inserted through conjunctiva and sclera intravitreally, 6 mm behind the limbus at the 1 o’clock position. The MV solution was injected directly into the vitreous body in a volume of 0.1 mL. One month following treatment, the behavioral changes were re-evaluated and both ophthalmoloscopic and ERG examinations were repeated.

### 2.6. Statistical Analysis

The in vitro assays were performed in triplicate. The data obtained was analyzed using GraphPad Prism 5 software (La Jolla, CA, USA). Differences between groups were determined using parametric assays, un-paired Student’s t–test or one-way analysis of variance (ANOVA). Differences with a probability of *p* ≤ 0.05 were considered to be significant.

## 3. Results

### 3.1. The Characteristic Features of CaMLCs Used in the Experiment

**Figure 1:**
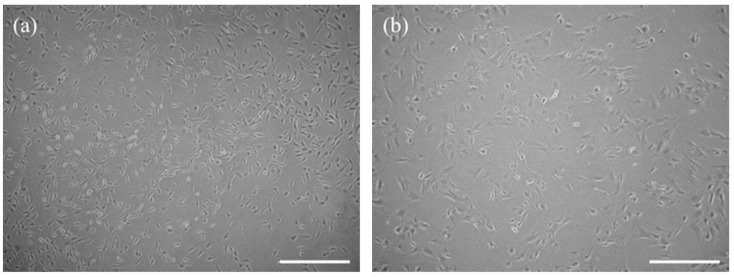
The morphology of canine Müller-like cells (CaMLCs). The cells were captured using an inverted microscope with phase contrast. Magnification was 50× (**a**, scale bar 500 µm) and 100× (**b**, scale bar 250 µm) and they exhibited features typical for glia-like cells.

**Figure 2:**
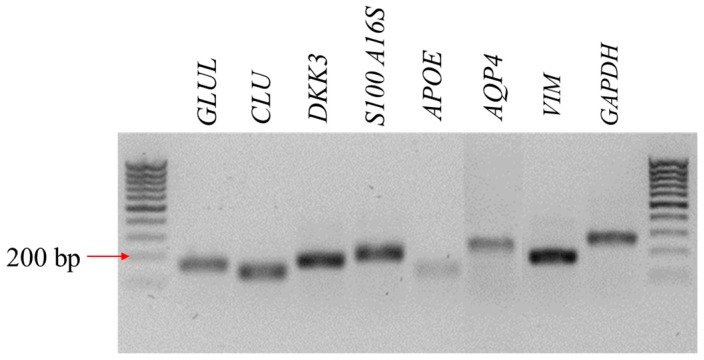
The electropherograms of PCR products identified based on their length (base pair/bp). The CaMLCs expressed specific markers, such as glutamine synthetase (*GLUL*), clusterin (*CLU*), dickkopf homolog 3 (*DKK*3), S100 calcium binding protein A16 (S100A16), apolipoprotein E (*APOE*), aquaporin 4 (AQP4) and vimentin (*VIM*), glyceraldehyde-3-phosphate dehydrogenase (*GAPDH*).

The Müller-like cells obtained from the canine retina were homogenous, with characteristic glia-like morphology and centrally located nuclei. They had broad lamellipodia and were symmetrical in shape. They formed a uniform layer and connected with each other by cellular projections (Figure 1). The CaMLCs used in the experiment expressed glutamine synthetase (Glul), clusterin (Clu), dickkopf homolog 3 (Dkk3), S100 calcium binding protein A16 (S100A16), apolipoprotein E (ApoE), aquaporin 4 (AQP4) and vimentin (Vim) (Figure 2), all typical markers for Müller cells.

### 3.2. The Evaluation of Biological Properties of hASC-MVs–In Vitro

**Figure 3:**
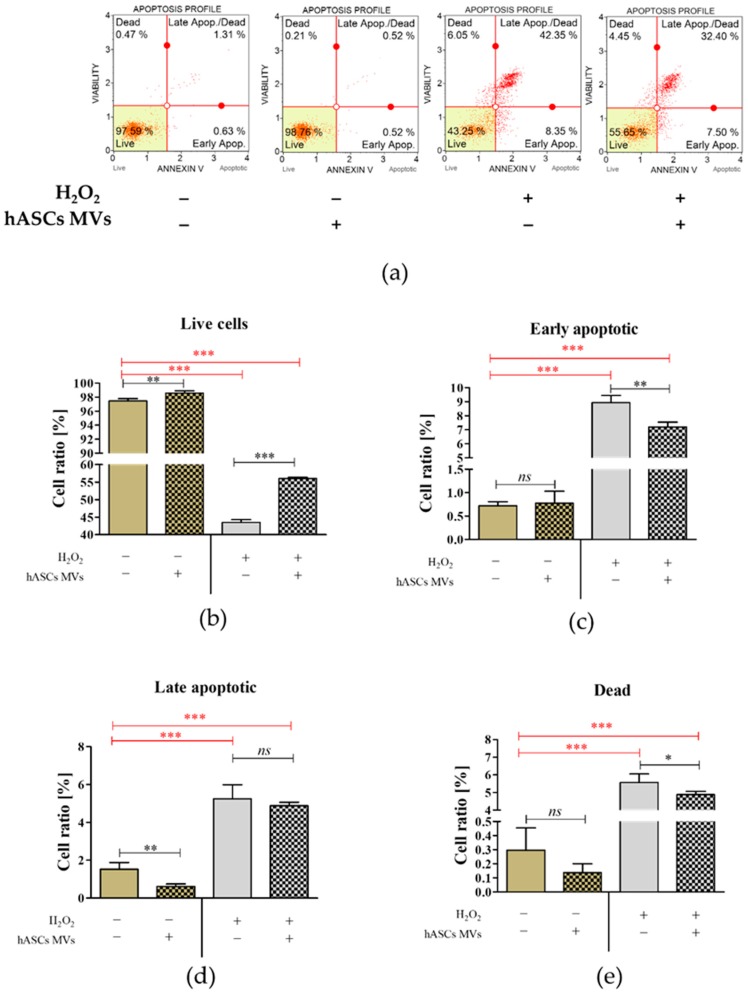
The influence of MVs on CaMLCs viability under both normal and oxidative stress conditions induced by H_2_O_2_.The analysis of cells viability was performed using Muse^®^ Annexin V and Dead Cell Assay Kit. The representative dot plots show the distribution of cells into four populations (**a**). The populations were determined based on the fluorescence of Annexin V and 7-AAD dye.—live (lower left quadrant; Annexin V −/7-AAD −), early apoptotic (lower right quadrant; Annexin V +/7-AAD −), late apoptotic (upper right quadrant; Annexin V +/7-AAD +), and dead cells (upper left quadrant; Annexin V +/7-AAD +). Comparative statistical analysis was performed in order to determine the influence of experimental condition on cells’ viability (**b**) and apoptosis profile (**c**,**d**). The presence of dead cells (nuclear debris) was also determined (**e**). An asterisk highlights the statistically significant difference (* *p* ≤ 0.05; ** *p* ≤ 0.01 and *** *p* ≤ 0.001) and differences between healthy cultures and cultures with induced oxidative stress are marked with red font. The results are expressed as mean ± S.D.

Analysis of CaMLCs’ viability demonstrated that MVs significantly improved health status, both in normal growth conditions and during oxidative stress. Oxidative stress significantly reduced the percentage of viable cells in CaMLCs, simultaneously increasing the number of early and late apoptotic cells. The addition of hASC-MVs in oxidative stress resulted in increased viability in the CaMLCs cultures. The addition of MVs to the H_2_O_2_ challenged CaMLC cultures significantly decreased the percentage of early apoptotic and dead cells, however it did not influence late-apoptotic subset distribution. (Figure 3).

**Figure 4:**
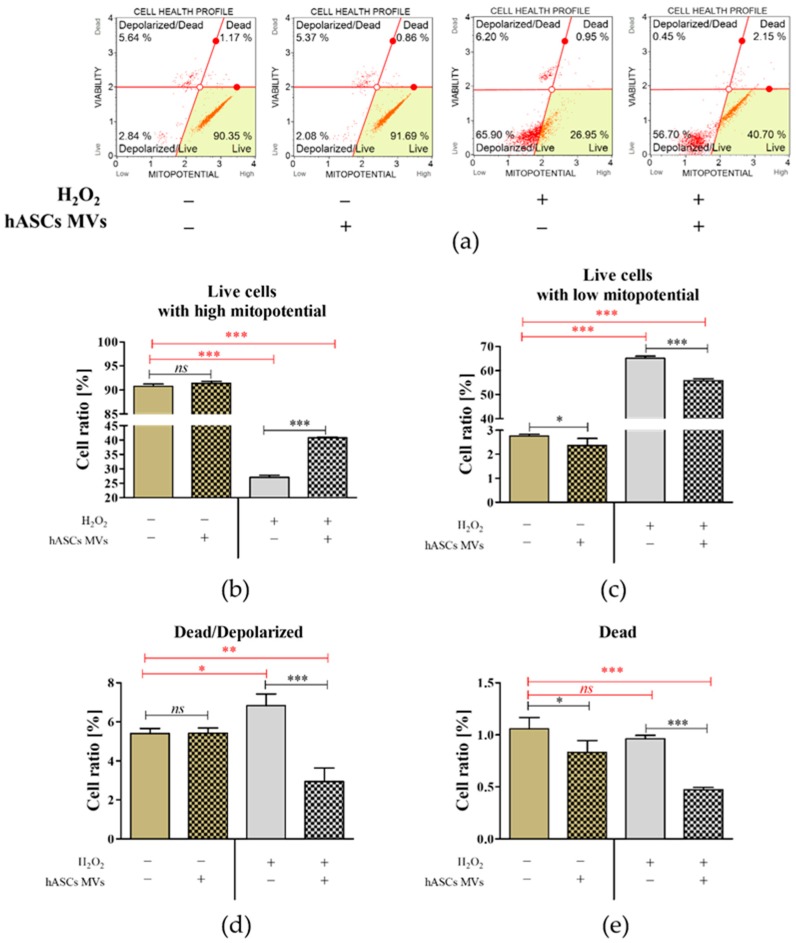
The influence of MVs on mitochondrial membrane potential in CaMLCs cultures both under normal and oxidative stress conditions induced using H_2_O_2_. The analysis was performed using the Muse^®^ Mitopotential Assay Kit. The representative graphs (**a**) show gated cells with quadrant marker providing data on four cell populations—live cells with depolarized mitochondrial membrane (lower left quadrant), live cells with intact mitochondrial membrane (lower right quadrant), dead/depolarized cells (upper left quadrant) and dead cells (upper right quadrant). The analysis was performed in triplicate, and the comparative statistic was performed (**b**–**e**). An asterisk marks a statistically significant difference (* *p* ≤ 0.05; ** *p* ≤ 0.01 and *** *p* ≤ 0.001). Differences between healthy cultures and cultures with induced oxidative stress are marked with red font. The results are expressed as mean ± S.D.

The significant decreased viability of cells under oxidative stress, was accompanied by their low metabolic activity manifested inter alia by low mitochondrial membrane potential (Figure 4). However, we noticed that introduction of hASC-MVs significantly increased the number of CaMLCs with high mitochondrial potential under oxidative stress. This was also associated with a reduced number of cells classified as live but with low mitochondrial potential, both noted in cultures treated with H_2_O_2_ under control conditions (Figure 4c). A significant increase of non-viable and non-viable depolarized CaMLCs was, also noted in cultures propagated under oxidative stress conditions and in the presence of MVs (Figure 4d,e).

**Figure 5:**
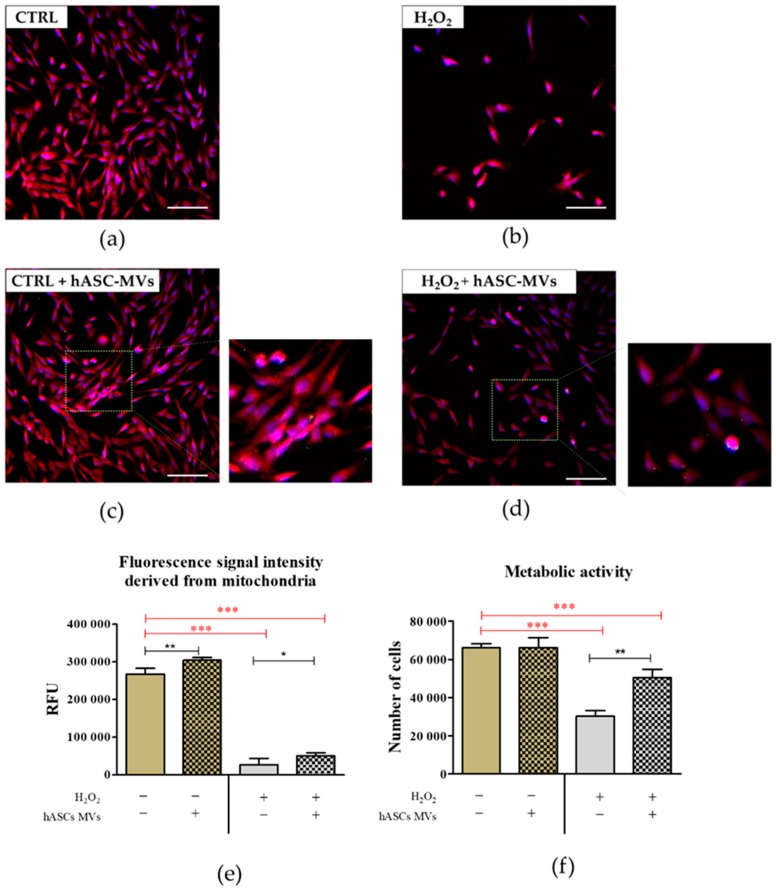
The analysis of mitochondrial morphology and activity, based on fluorescence signal intensity and analysis of CaMLCs metabolic activity. The control cultures were propagated using typical growth medium (**a**) as well as under oxidative stress conditions (**b**). The morphology of cells and mitochondria activity was determined also in cultures with introduced hASC-MVs (**c**,**d**, respectively). The hASC-MVs were labeled with green PKH67 dye, notice in cultures as green-yellow dots (magnified square fields). Images were analyzed under 60-fold magnification (scale bar 200 µm). The insensitivity of fluorescence derived from stained mitochondria was measured using ImageJ software (**e**). Metabolic activity was measured using Alamar Blue assay (**f**). The obtained results were used for statistical analysis. The significant differences were marked using an asterisk (* *p* ≤ 0.05; ** *p* ≤ 0.01 and *** *p* ≤ 0.001). Differences between healthy cultures and cultures with induced oxidative stress are additionally marked with red font. The results are expressed as mean ± S.D.

The results of an Alamar blue assay showed that the addition of hASC-MVs significantly improved the metabolic activity of CaMLCs under oxidative stress conditions. The hASC-MVs also increased the metabolism of cells in healthy cultures, but this change was not statistically significant (Figure 5). These observations are in a good agreement with the images showing mitochondrial morphology and analysis of the fluorescence signal intensity derived from mitochondria is also in line with the results indicating mitochondrial membrane potential.

**Figure 6:**
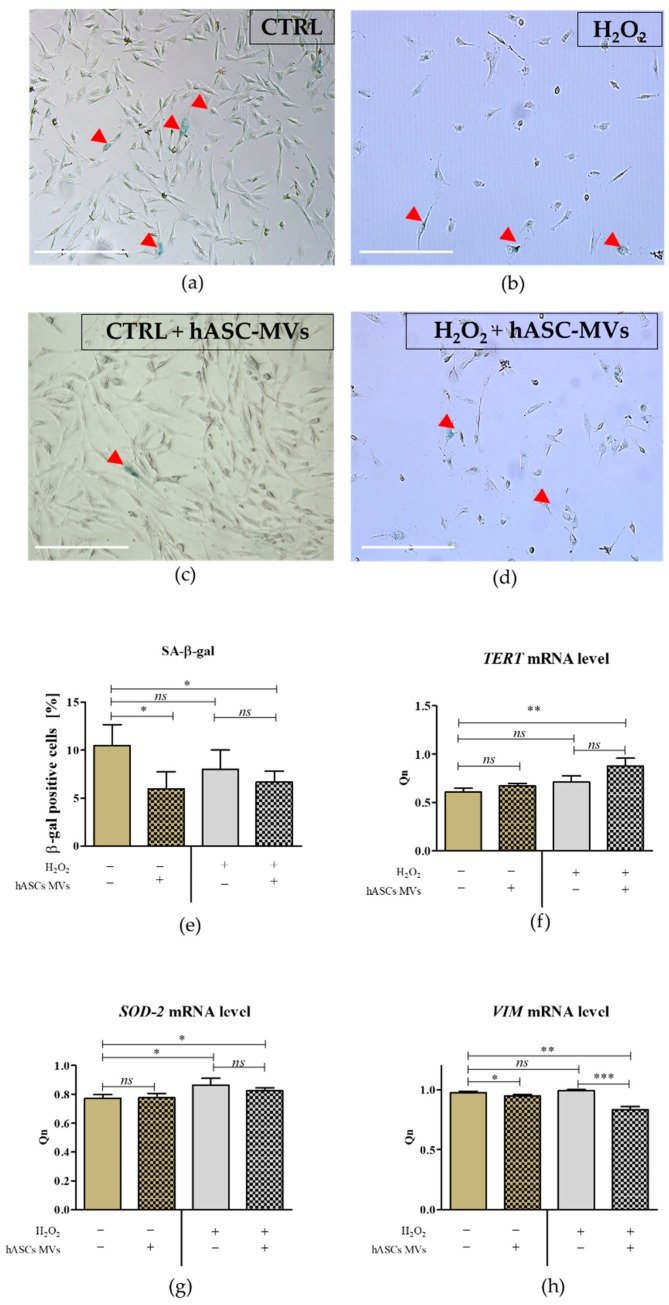
The analysis of the senolytic action of MVs both in control and oxidative stress induced cultures (**a**–**d**). The β-galactosidase positive cells are marked with red arrows. The number of cells was counted using ImageJ software and analyzed statistically (**e**). The expression of markers associated with cell senescence was measured using RT-qPCR. Following transcripts were measured: telomerase reverse transcriptase (*TERT;* **f**); mitochondrial superoxide dismutase 2 (*SOD-2*; **g**) and vimentin (*VIM*; **h**). The transcripts levels were normalized (Qn) to the reference gene (here: Glyceraldehyde-3-Phosphate Dehydrogenase; GAPDH). The normalized values (Qn) were analyzed using statistical tests. The significant differences are marked with asterisks (* *p* < 0.05; ** *p* < 0.01 and *** *p* < 0.001) and differences between healthy cultures and cultures with induced oxidative stress are marked in red font. The results are expressed as mean ± S.D.

The analysis of senescence marker β-galactosidase showed that MVs decrease the percentage of senescent cells in CaMLCs cultures, with the most significant senolitic effect of MVs being noted under normal conditions. The analysis of mRNA levels for telomerase reverse transcriptase (TERT) and mitochondrial superoxide dismutase 2 (SOD-2) showed that MVs did not alter their expression in CaMLCs cultures. Nevertheless, both TERT and SOD-2 were upregulated in cultures in which oxidative stress had been induced (Figure 6).

**Figure 7:**
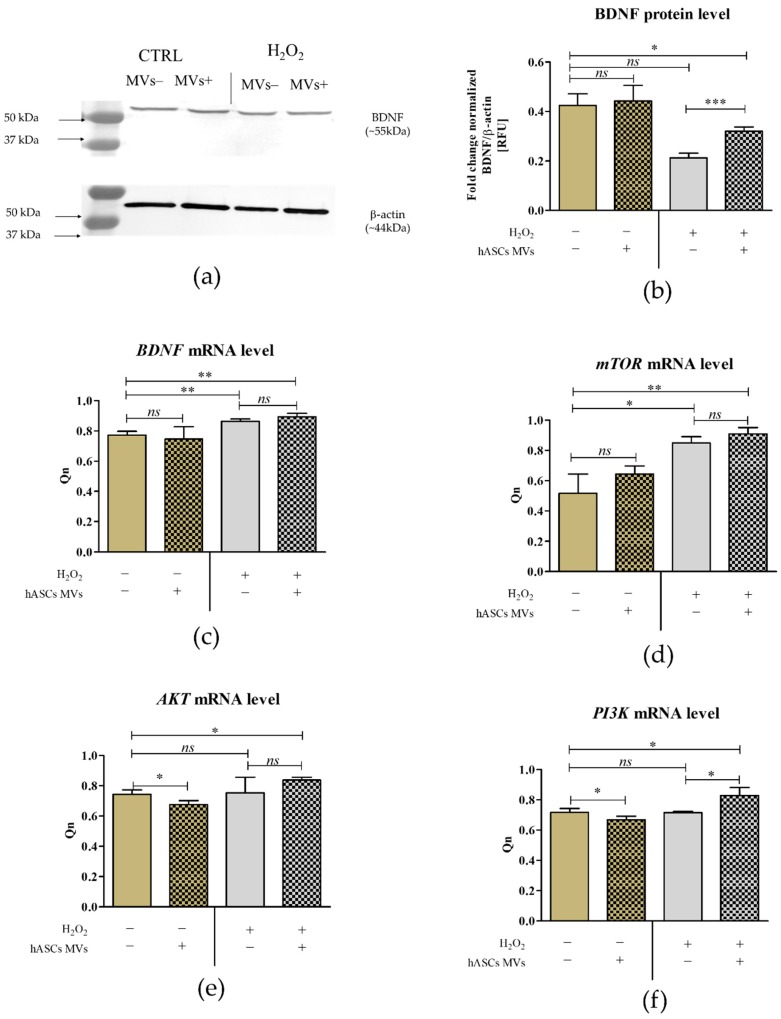
The influence of MVs on the expression of brain derived neurotrophic factor (BDNF) and elements of PI3K/Akt/mTOR signaling pathway. BDNF accumulation in cells was determined using the Western blot technique (**a**). The densitometry analysis was performed using ImageLab software, the amount of accumulated protein was determined in relation to the β-actin (**b**). The mRNA levels for *BDNF* (**c**), *mTOR* (**d**) *AKT* (**e**) and *PI3K* (**f**) were established using RT-qPCR, and quantitative analysis was performed in reference to *GAPDH*. The obtained results i.e., normalized values (Qn) were tested using statistical methods. An asterisk marks statistically significant differences (* *p* ≤ 0.05, ** *p* ≤ 0.01and *** *p* ≤ 0.001) and differences between healthy cultures and cultures with induced oxidative stress are marked in red font. The results are expressed as mean ± S.D.

The BDNF expression determined on mRNA and protein levels (the concentration of intracellularly accumulated protein) was stable in CaMLCs maintained in the control cultures. Oxidative stress in CaMLCs was associated with a significant increase of mRNA for BDNF, but the addition of hASC-MVs did not influence the transcript level. BDNF concentration decreased after oxidative stress inducement, but treatment with MVs significantly (*p* < 0.05) improved its accumulation in CaMLCs. The profile of the mTOR gene expression was similar to the mRNA expression of BDNF. It appeared that oxidative stress induced in CaMLCs was also associated with increased levels of mTOR transcripts, but again the MVs did not alter the expression of mTOR. It was also noted that the mRNA levels for Akt and PI3K in CaMLCs appeared to have similar patterns of expression. Generally, the addition of MVs to healthy cultures significantly decreased the transcripts levels, while under oxidative stress it caused an increase of mRNA levels for Akt and caused a significant increase for PI3K. The expression of Akt and PI3K was significantly increased in cultures treated with hASC-MVs in comparison with the control cultures (Figure 7).

### 3.3. The Evaluation of Biological Properties of hASC-MVs–In Vivo

#### 3.3.1. Behavioral Observations

Eight of ten dogs demonstrated an improvement in behavior in both dark and dim lighting conditions. In the remaining one dog the owners did not notice any change. The observations before and after therapy in both dark and dim lighting conditions are described in Table 2.

In eight of the test dogs maze testing revealed that following hASC-MVs therapy these dogs expressed less hesitation in the dark and no hesitation in dim lighting conditions. The dog which showed no improvement was the five year old crossbred with suspected PRA, the second was the fifteen year old cross-bred with retinal degeneration due to inflammation associated with Borrelia infection.

#### 3.3.2. Ophthalmic Examination

**Figure 8:**
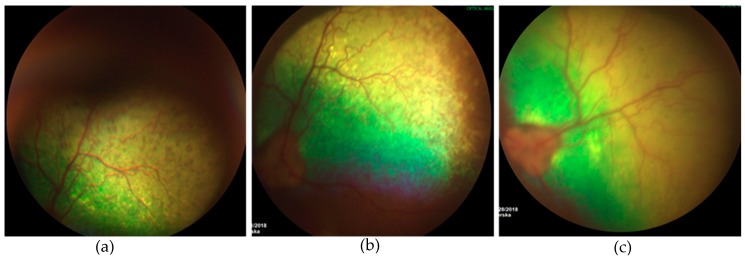
Fundus photographs before (**a**) and following hASC-MVs injection at one (**b**) and three-months (**c**). A noticeable decrease in tapetal pigmentation was observed, but areas of focal hyperreflectivity were noted nearer to the optic disc.

Funduscopic changes were not seen during the first month following treatment. Representative image of the analysis is presented in the Figure 8. Three year old Kelpie with a retinopathy of unknown origin, noticeable fundus changes were found following treatment. Initially there had been focal areas of hyperreflectivity as well as areas of pigmentation in the tapetal fundus. After three months we observed less pigment in the peripheral tapetal fundus, although some areas of hypereflectivity had appeared nearer to the optic disc (Figure 8). 

#### 3.3.3. Electroretinography (ERG) Post hASC-MV Administration

**Figure 9:**
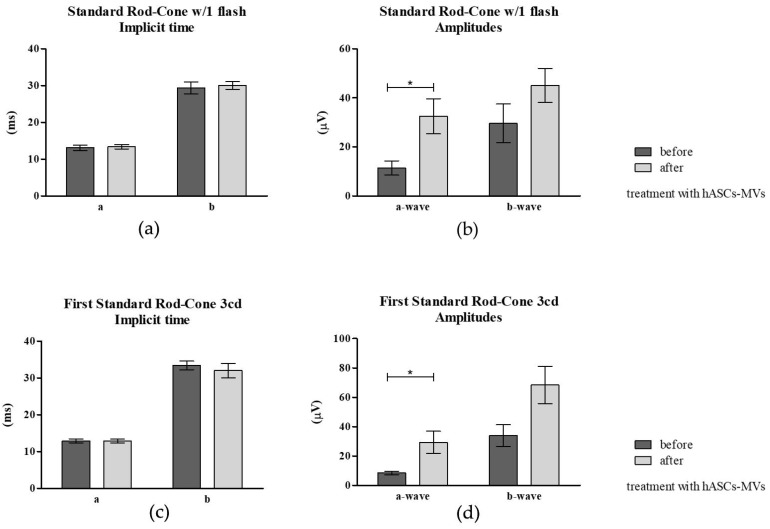

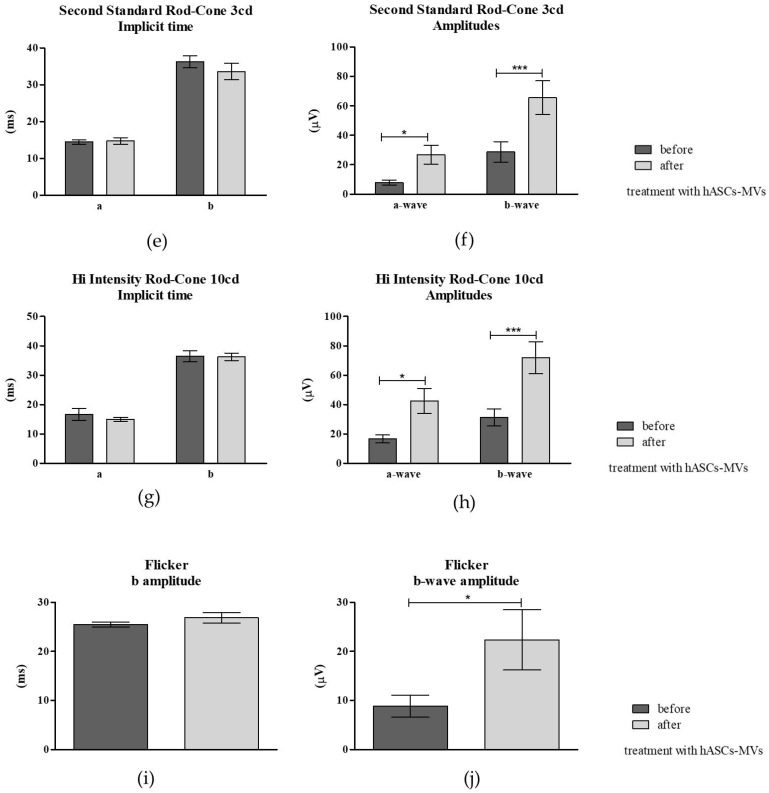
The results of ERG measurements. Mean values of ERG recordings obtained for nine dogs. The analysis included comparison of implicit times as well as a- and b-wave amplitudes before and after hASC-MVs implantation. The analysis was based on determination of following parameters: Standard Rod-Cone w/1 flash using implicit time (**a**) and amplitudes (**b**); First Standard Rod-Cone 3cd with implicit time (**c**) and amplitudes (**d**); Second Standard Rod-Cone 3cd with implicit time (**e**) and amplitudes (**f**); Hi Intensity Rod-Cone 10cd with implicit time (**g**) and amplitudes (**h**); Flicker b amplitude implicit time (**i**) and Flicker b-wave amplitude (**j**). Statistically significant differences are indicated with asterisks (*). The statistically significant differences were determined at *p* ≤ 0.05 (*) and *p* ≤ 0.001 (***). The results are expressed as mean ± S.D.

Short protocol electroretinography was completed under the same conditions as those prior to the MV treatment. The a- and b-wave amplitudes were measured from the baseline to the first trough and from that trough to the next positive peak. Implicit times were measured from the stimulus to the positive peak. For the flicker tests, the frequency domain amplitude of the first harmonic was extracted. Analysis showed that both the a and b-wave amplitudes significantly increased after MV injection (Figure 9a). Analysis of standard rod cone w1/flash demonstrated an increase in the a- and b-wave amplitude after treatment, although the significant improvement was observed only for the a-wave. (Figure 9b–d). The first and second standard rod-cone 3cd, as well as the high intensity rod- cone 10cd demonstrated that MV therapy did not influence the implicit time, but significantly improved the a- and b-wave amplitudes (Figure 9e). The flicker analysis revealed that the b-wave implicit time increased, but the difference was not statistically significant. There was a significant increase in the b-wave amplitude after hASC-MVs injection.

**Figure 10:**
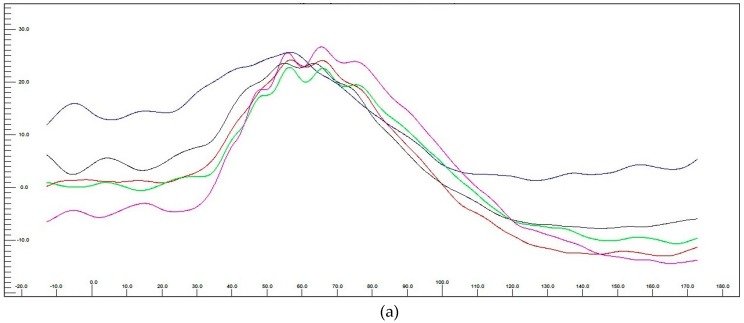

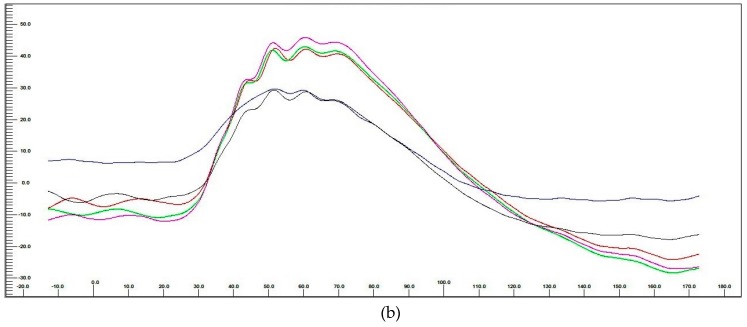
Representative electroretinography traces presenting dark adaptation before (**a**) and after (**b**) MV injection. There is a significant difference in the amplitude changes during the dark adaptation. Each color-marked trace represents a time-delayed recording made during 20 min of ERG examination consisting of 5 measurements every 4 min.

According to the Dog Diagnostic Protocol (long protocol) performed in one of ten dogs a- and b-wave implicit time remained stable whereas a- and b-wave amplitude increased in rod and rod cones responses (Table 3) Additionally improvement in the dynamic process of dark adaptation was seen (Figure 10).

## 4. Discussion

This study has demonstrated for the first time the effect of hASC-MVs on CaMLCs in both normal and oxidative stress conditions. We examined the influence of hASC-MVs on CaMLCs viability, proliferative and metabolic activity, as well as the expression of BDNF. In addition the role of hASC-MVs on mitochondrial membrane potential, the expression of senescence markers as well as the oxidative stress markers have been determined. The possible therapeutic potential of hASC-MVs was evaluated in dogs with retinal degeneration and to the best of our knowledge this is the first report showing a potential application of MV’s in the treatment of canine retinal degenerations. 

An in vitro model of CaMLCs was used to evaluate the biological activity of MVs obtained from hASCs. These CaMLCs presented as a glial-like morphotype and were identified using markers typical for Muller-like cells [38,39,40]. The results indicated that MVs significantly improve the viability of these cells, both under normal growth conditions as well as under oxidative stress. The results are in accord with the Eldh et al. study (2010) which demonstrated that exosomes derived from mast cells under oxidative stress may have the ability to induce tolerance to oxidative stress in recipient cells [41]. Further it was shown that MVs derived from human Wharton Jelly mesenchymal stromal cells (hWJMSCs) may have therapeutic potential and could protect kidney cells against renal ischemia/reperfusion injury (IRI) by mitigating the oxidative stress [42]. In our study we have demonstrated that MVs derived from hASCs may alleviate the oxidative stress effect in CaMLCs by improving their mitochondrial potential. This is also in good agreement with studies of Yu et al. (2015) who showed that exosomes derived from mesenchymal stromal cells may contribute to increased survival, reduced apoptosis and may preserve mitochondrial membrane potential in neonatal cardiomyocytes cultured under hypoxic conditions [43]. Furthermore, our study also indicated on a positive senolytic action of hASC-MVs toward canine Müller-like cells. The senolytic effect of hASC-MVs was related to decreased expression of senescence associated markers i.e., β-galactosidase and vimentin in CaMLCs, but also with their increased metabolic activity Obtained results correspond with the studies showing that human induced pluripotent stem cell-derived exosomes may have an anti-senescence effect on high glucose-injured human umbilical vascular endothelial cells (HUVECs) [44]. Our study has also demonstrated that MVs may contribute to the greater functionality of CaMLCs in oxidative stress conditions, improving their production of BDNF. It has been previously shown that hASCs can provide neurotrophic growth factors, particularly BDNF, to injured nerves [45]. Additionally, Martins et al. (2017) showed that the secretome of mesenchymal stromal cells is able to promote axonal outgrowth in central nervous system (CNS) neurons and that this effect may be mediated by BDNF [46].The results of in vitro studies clearly demonstrate that vesicles derived from ASC are able to regulate metabolism, cell senescence and apoptosis of CaMLCs. These cytophysiological features greatly affect the therapy outcome. Indeed, MSC-derived vesicles were shown to have positive impact on tissue repair via various pathways, mainly those associated with cell death and senescence, for instance resulting from ischemic injury [47]. It is also emphasized that therapeutic effect of mesenchymal stem cells relies on their paracrine effect and immunomodulatory properties [47,48], due to this fact various cell-free therapies based on MVs implantation are being evaluated [49]. 

In our study, we were interested whether the hASCsMVs may improve retinal function. The clinical part of the study was completed in canine patients with different types of retinal degeneration. In eight of the ten treated animals we have shown that the intravitreal injection of MVs in dogs with retinal degeneration of both of presumed genetic and acquired etiologies lead to a reversal of adverse behavioral problems and an improvement in maze performance. Our data corresponds with previous results showing a positive effect of bone marrow derived mesenchymal stem cells in the treatment of human retinitis pigmentosa patients. Siqueira et al. (2015) demonstrated that quality of life in these patients was transiently but significantly improved [50].

The dogs included in our research already had advanced signs of retinal degeneration, demonstrating blood vessel attenuation and varying degrees of tapetal hyperreflectivity. The ophthalmoscopic changes observed three months after treatment are not dramatic, but they seemingly parallel the improvements in both the behavioral and electroretinographic results. Simple screening electroretinography is routinely used in the investigation of retinal degenerations in animal models [51] and in short protocol electroretinography only gross retinal function can be determined. We were able use the technique to demonstrate that intra-vitreal MVs implantation improved retinal functionality. Increases in the a- and b-wave amplitudes were recorded, indicating improvement in photoreceptor and Muller cell functions respectively. The Dog Diagnostic Protocol performed in one dog revealed also the improvement in process of dark adaptation of rod photoreceptors. The ERG results obtained in particular animals correlated with both their improved behavioral activity and performance in maze testing. However, further studies are required to substantiate our preliminary results and to assess the potential value of MVs implantation in the long term outcomes. 

## 5. Conclusions

The results of this study indicate a potential future use of MVs derived from adipose-tissue stromal cells in the treatment of degenerative canine retinal disorders. MVs contributed to increased viability and metabolic activity of Müller like-cells in vitro, whilst their intra-vitreal administration significantly improved photoreceptor and Müller cell function. The results also shed promising light on application of MVs-based therapy in the treatment of retina degeneration. 

## Figures and Tables

**Table 1:** Characteristics of animals used in the experiment.

Animal	Sex	Age (Years)	Breed/Type	Retinopathy
**1**	female	12	Crossbred	Suspected progressive retinal atrophy (PRA)
**2**	male	5	Crossbred	Suspected progressive retinal atrophy (PRA)
**3**	female	7	Yorkshire Terrier Cross	Suspected progressive retinal atrophy (PRA)
**4**	male	15	Crossbred	Associated with *Borrelia sp.* infection
**5**	female	2	Border Collie	Associated with systemic infection of unknown etiology
**6**	female	10	Crossbred	Suspected progressive retinal atrophy (PRA)
**7**	female	7	Welsh Terrier	Suspected progressive retinal atrophy (PRA)
**8**	male	7	German Shepherd Dog	Suspected progressive retinal atrophy (PRA)
**9**	male	12	Continental Spaniel	Suspected progressive retinal atrophy (PRA)
**10**	female	3	Australian Kelpie	Unknown

**Table 2:** The behavioral features of animals before and after therapy with hASC-MVs.

Features	Before Therapy	After Therapy	*p* Value
**General behavior**	Anxiety, reluctance towards with the handler, aggression with other dogs, problems negotiating obstacles, difficulties in dark rooms, visible stress and frustration.	Calmer, more confident and more tolerant with other dogs. Still a need to adapt after entering dark room but the time needed was shorter. Training was easier on days without very bright light.	*p* < 0.05
**Tracking**	Lack of motivation, problems with changes in direction and easily distracted.	Calmer, very precise, focused on the line of scent, no frustration when the track changed, low stress.	*p* < 0.05
**Other relevant comments**	Problems with catching objects and judging distances	Slow improvement in catching objects, more dynamic but still problems in dim lighting at night.	*p* < 0.05

**Table 3:** The results of ERG analysis using long protocol. Study performed on the Kelpie dog with retinal degeneration of unknown etiology.

Parameter		Before hASC-MVs Treatment	After hASC-MVs Treatment
Rod1	a (ms)	5.6	3.9
	b (ms)	56.7	51.8
	a-wave (µV)	3.3	0.7
	b-wave (µV)	12.8	23.5
Rod2	a (ms)	14.4	16.5
	b (ms)	55	51.9
	a-wave (µV)	0.6	1.3
	b-wave (µV)	20.3	49.9
Rod3	a (ms)	20	3
	b (ms)	56.7	51.9
	a-wave (µV)	0.8	2.2
	b-wave (µV)	23.3	49.9
Rod4	a (ms)	13.9	18.4
	b (ms)	56.4	51
	a-wave (µV)	0.6	0.4
	b-wave (µV)	23.4	52.8
Rod5	a (ms)	2.2	20.2
	b (ms)	56.2	51.9
	a-wave (µV)	1.3	1.4
	b-wave (µV)	31.1	56
Standard rod and cone responses	a (ms)	13.3	12.1
	b (ms)	33.2	30.9
	a-wave (µV)	33.9	43.1
	b-wave (µV)	79.9	105
High intensity rod and cone response	a (ms)	12.8	11.2
	b (ms)	32.8	30.5
	a-wave (µV)	41.6	48
	b-wave (µV)	87.1	101.9
Cone response	a (ms)	9.1	7.7
	b (ms)	26.1	25.5
	a-wave (µV)	5.6	4.4
	b-wave (µV)	17.2	13
Cone flicker response	b (ms)	25.2	25.2
	b-wave (µV)	34.2	29.2

## Supplementary Materials

The following are available online at https://www.mdpi.com/2077-0383/8/4/510/s1, Table S1: Sequences and description of primers used for qPCR.
