# Supplementary material for: Intra-Vitreal Administration of Microvesicles Derived from Human Adipose-Derived Multipotent Stromal Cells Improves Retinal Functionality in Dogs with Retinal Degeneration
Source: J Clin Med. 2019 Apr 13;8(4):510. doi: 10.3390/jcm8040510 (PMC6518198; doi:10.3390/jcm8040510)
Supplement: Supplementary file 1 [file jcm-08-00510-s001.pdf]

Table 1. Sequences and description of primers used for qPCR.

Gene name	Gene abbreviation	Primer	Sequence 5'-3'	Loci	Amplicon lenght [bp]	Accesion no.
Glutamate-ammonia ligase	Cl-GLUL	F:	CCATGTTTCGCGACCCTTTC	491-510	153	NM_001002965.2
		R:	CCATTCCAAACCAGGGGTGT	643-624		
Clusterin	Cl-CLU	F:	ATACACCAGGCTCAACAGGC	801-820	124	NM_001003370.1
		R:	AACCTGTGGAGTTGTGACGG	924-905		
Dickkopf WNT signaling pathway inhibitor 3	Cl-DKK3	F:	ACCCTCAACGAGATGTTCCG	136-155	171	NM_001284475.1
		R:	CCTGGTTTCCGTGTTGGTCT	306-287		
S100 calcium binding protein A16	Cl-S100A16	F:	ACAGATTCTGGGAGCGAAGC	73-92	190	XM_861080.5
		R:	CTTCTCTGGAGCGTTGTCCA	262-243		
Apolipoprotein E	Cl-ApoE	F:	GGGGCAGGGGGAACATTAT	30-48	120	XM_533644.6
		R:	CAGCCCACAGAACCTTCATCT	149-129		
Aquaporin 4	Cl-AQP4	F:	AACCCAATCAGACAAGTGGC	46-65	192	XM_850363.5
		R:	GCCTTCCAGAAAGCTTGAGTC	237-217		
Vimentin	Cl-VIM	F:	GCAGGATTGCTCTGCCTCTT	1363-1382	184	NM_001287023.1
		R:	GGCAAGCTTCACTCAAGGTC	1546-1527		
Mitochondrial superoxide dismutase 2	Cl-SOD2	F:	CGCTGGAGAAGGGTGACATT	224-243	156	XM_533463.6
		R:	CACGTTTGATGGCTTCCAGC	379-360		
Telomerase reverse transcriptase	Cl-TERT	F:	CGCGTTGTTTCTGGATCTGC	2969-2988	163	NM_001031630.1
		R:	CGCGATAACACGGAGGAAGA	3131-3112		
Brain-derived Neurotrophic Factor	Cl-BDNF	F:	ACCGTCCTCGAAAAAGTCCC	712-731	151	XM_005633266.3
		R:	CGTACGACTGGGTAGTTCGG	862-843		

Mechanistic target of Rapamycin Kinase	Cl-mTOR	F:	CAACCCGATGGCCAGCATTA	4053-4072	184	XM_005618004.3
		R:	GGGGTCATCCTTGTTCGTGT	4236-4217		
Phosphatidylinositol-4-phosphate 3-kinase	Cl-PI3K	F:	GCCAAAGGGATGCCCTAAGT	6077-6096	182	XM_014111226.2
		R:	GGTGTGAATCCGAAGTCCGT	6258-6239		
AKT serine/threonine kinase 2	Cl-AKT2	F:	GCCTCTTCGAGCTCATCCTC	1323-1342	191	XM_014114088.2
		R:	CTTCTGTACCACGTCCTGCC	1513-1494		
Glyceraldehyde-3-Phosphate Dehydrogenase	Cl-GAPDH	F:	GATTGTCAGCAATGCCTCCT	491-510	198	NM_001003142.2
		R:	GTGGAAGCAGGGATGATGTT	688-669		
